# Supplementary material for: Causal interplay between lactose intolerance and gut microbiota: a combined bidirectional Mendelian randomization and in vivo validation study
Source: Front Nutr. 2026 Jun 1;13:1803337. doi: 10.3389/fnut.2026.1803337 (PMC13265576; doi:10.3389/fnut.2026.1803337)
Supplement: Supplementary file 1 [file Data_Sheet_1.zip › supplementary materials/Forward/leave one out cross validation/ebi-a-GCST90027636.finngen_R12_E4_LACTONAS.pdf]

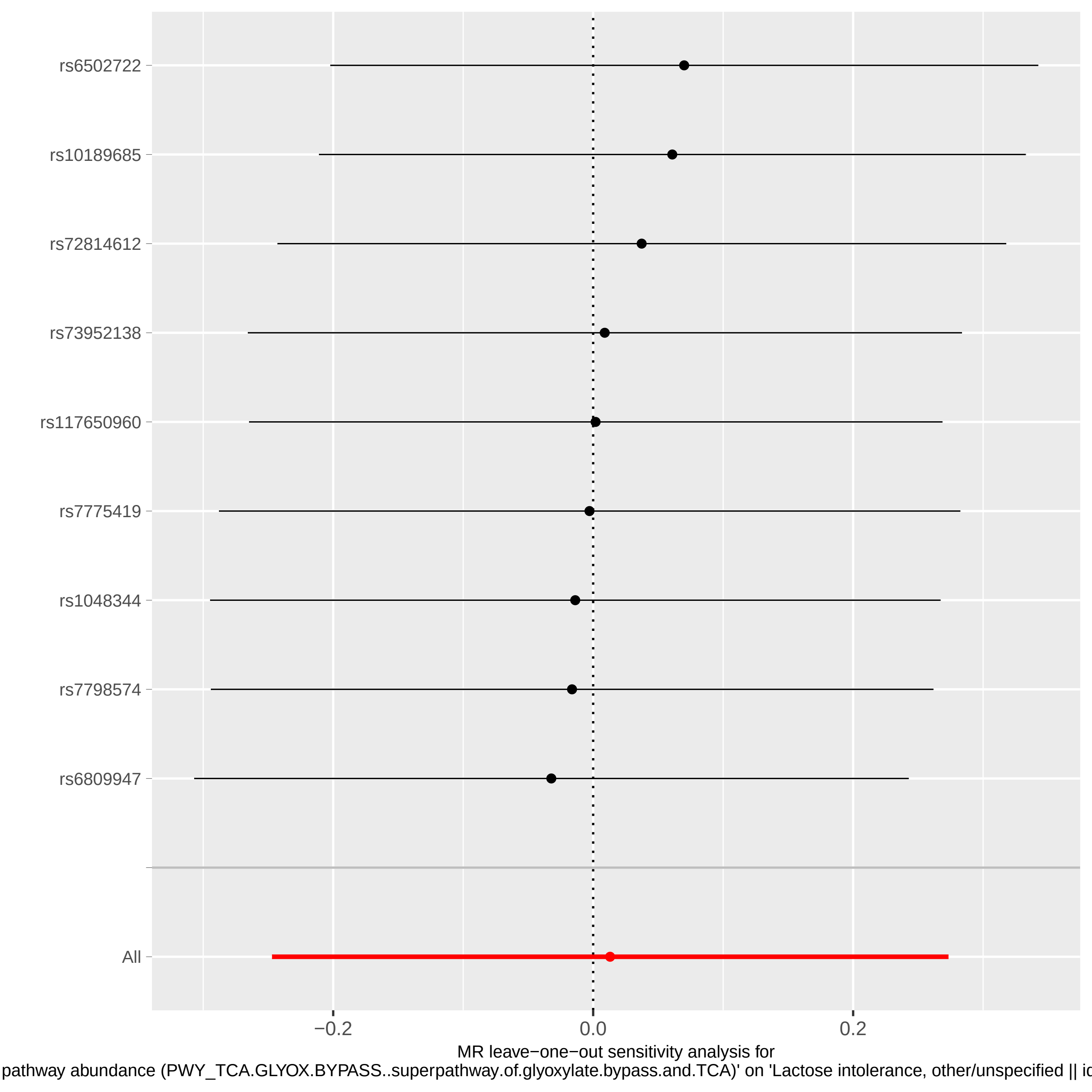
rs6502722
rs10189685
rs72814612
rs73952138
rs117650960
rs7775419
rs1048344
rs7798574
rs6809947
All
−0.2
0.0
0.2
MR leave−one−out sensitivity analysis for
pathway abundance (PWY_TCA.GLYOX.BYPASS..superpathway.of.glyoxylate.bypass.and.TCA)' on 'Lactose intolerance, other/unspecified || id
